# Comparative Analysis of Modified BT Shunt and Central Shunt in Pediatric Patients

**DOI:** 10.21470/1678-9741-2023-0376

**Published:** 2024-05-13

**Authors:** Mustafa Yilmaz, Başak Soran Turkcan, Ata Niyazi Ecevit, Yasemin Özdemir Şahan, Atakan Atalay

**Affiliations:** 1 Department of Pediatric Cardiovascular Surgery, Ankara Bilkent City Hospital, Ankara, Turkey; 2 Department of Pediatric Cardiology, Ankara Bilkent City Hospital, Ankara, Turkey

**Keywords:** Sternotomy, Cardiopulmonary Bypass, Hospital Mortality, Heart Failure, Infant, Newborn, Child, Congenital Heart Defects

## Abstract

**Introduction:**

Cyanotic congenital heart diseases constitute 40-45% of all congenital heart diseases. In patients who are not suitable for primary repair, modified BT (MBT) shunt and central shunt (CS) procedures are still frequently used.

**Methods:**

This study included 62 pediatric patients who underwent MBT shunt or CS via median sternotomy. Patients’ demographic, echocardiographic, operative, and postoperative data were collected retrospectively. The patients were classified as single ventricle and bi-ventricle according to their cardiac anatomy, and the presence of prematurity and heterotaxy was noted. Procedure details of the patients who underwent endovascular intervention prior to the surgery were investigated, and operation data were accessed from the surgery notes. Data regarding postoperative follow-ups were obtained and comparatively analyzed.

**Results:**

Of the total 62 patients, 32 (51.6%) were newborns and 16 (25.8%) had a body weight < 3 kg. MBT shunt was applied to 48 patients (77.4%), while CS was applied to 14 patients (22.6%). There was no significant difference between the two surgical procedures in terms of requirement for urgent shunt or cardiopulmonary bypass, additional simultaneous surgical intervention, need for high postoperative inotropes, and in-hospital mortality (P>0.05). The rate of congestive heart failure in patients with in-hospital mortality was determined as 66.7% and it was significantly higher than in patients without heart failure (P<0.001).

**Conclusion:**

MBT shunt and CS are still frequently used in cyanotic patients. The use of small-diameter shunts, particularly when centrally located, can prevent the onset of congestive heart failure and lower mortality.

## INTRODUCTION

**Table:** 

Abbreviations, Acronyms & Symbols	
CI	= Confidence interval
CPB	= Cardiopulmonary bypass
CS	= Central shunt
IQR	= Interquartile range
MBT	= Modified BT
PDA	= Patent ductus arteriosus
PTFE	= Polytetrafluoroethylene
SD	= Standard deviation
VSD	= Ventricular septal defect

Cyanotic congenital heart diseases constitute 40-45% of all congenital heart diseases^[1]^. Thanks to developing technology, improved intensive care services, and surgical techniques refined over many years of experience, numerous cyanotic heart diseases can be primarily corrected in the neonatal or early infant period. However, a significant number of cyanotic patients are not suitable for primary repair in these periods. These cyanotic patients, who may have a single or biventricular anatomy, require well-developed pulmonary arteries to proceed through the palliation stages or to be suitable for complete correction, respectively^[2]^. For this purpose, systemic-pulmonary artery shunts are still frequently used.

Systemic-pulmonary artery shunts have undergone significant changes since their first application. Recently, the most commonly used modification is the modified BT (MBT) shunt, which was described by de Leval et al.^[3]^ in 1975, in which artificial vascular grafts made of polytetrafluoroethylene (PTFE) are interposed between the subclavian and pulmonary arteries. Thanks to these artificial grafts, shunt procedures can be performed both from the aorta itself and all epiaortic branches to the entire pulmonary artery bed.

The median sternotomy approach has gained popularity in systemic-pulmonary artery shunt procedures in recent years. This approach generally enables to reach the entire pulmonary artery bed and all aortic segments. This is especially important in patients whose right and left pulmonary arteries are underdeveloped and are not suitable for anastomosis. However, in some of these patients, the main pulmonary artery is well developed and suitable for shunting. In these cases, some clinics prefer to interpose a central shunt (CS) between the ascending aorta and the main pulmonary artery instead of MBT shunt^[4]^.

Although performing systemic-pulmonary artery shunts are typically straightforward, they can cause serious changes in flow dynamics after the procedure^[5]^. This situation may cause decompensated heart failure, requiring long-term use of inotropic agents and staying in intensive care for longer time than usual, or even death. For these reasons, it is necessary to closely monitor the clinical results of MBT shunt and CS procedures and to modify surgical strategies according to these results.

## METHODS

The sample of this study consisted of 62 patients who underwent MBT shunt or CS via median sternotomy between January 2019 and August 2023. Patients who underwent hypoplastic left heart stage 1 palliation were not included in the study because the procedure was complex and required long myocardial and mesenteric ischemia times.

Demographic, echocardiographic, operative, and postoperative data of the patients were collected retrospectively. Patients were classified as single ventricle and bi-ventricle according to their cardiac anatomy, and the presence of prematurity and heterotaxy was noted. Procedure details of the patients who underwent endovascular intervention prior to the surgery were investigated, and operation data were accessed from the surgery notes. Data regarding postoperative follow-ups were obtained by scanning digital and written documents.

The aims of the study were to determine the factors that may cause postoperative morbidity for MBT shunt and CS, as well as to reveal the factors which cause in-hospital mortality in all systemic-pulmonary artery shunt patients.

### Surgical Technique

Patients who were not suitable for endovascular patent ductus arteriosus (PDA) stenting or who had complications during or after the procedure were referred to surgery. Hemodilution in patients was achieved by loading of 20-30 cc/kg saline within half an hour before the operation. Median sternotomy was preferred as the standard clinical approach in all patients. After the complete resection of thymus, the pericardium was opened and the brachiocephalic artery and right and left pulmonary arteries were dissected. If the diameter of the widest of the right or left pulmonary arteries was < 3.5 mm, our clinical approach was to perform CS to the main pulmonary artery. In MBT shunt, 3.5 mm PTFE graft was preferred in patients < 3.5 kg, 4 mm in patients between 3.5 and 5 kg, and 5 mm in patients weighing > 5 kg. On the other hand, in CS, a 3.5 mm graft was used for patients < 4 kg, a 4 mm graft was used for patients weighing between 4 and 6 kg, and a 5 mm graft was used for patients weighing > 6 kg. After heparinization, a C-clamp was placed on the right pulmonary artery to check whether the patient could tolerate the procedure. If the patient tolerated clamp occlusion, the clamp was removed from the pulmonary artery and placed to include the proximal brachiocephalic artery. Following this, all adventitial tissues that could disturb the anastomosis were resected, and an arteriotomy was performed from the lower aspect of the brachiocephalic artery. Using 7/0 polypropylene suture, the PTFE vascular graft appropriate for the patient was anastomosed to the arteries in end-to-side fashion, using a continuous suturing technique. The C-clamp was removed from the brachiocephalic artery, the flow was checked, and then an obliquely angled cross-clamp was placed on the PTFE graft. Meanwhile, anastomotic bleeding was checked. Subsequently, a C-clamp was placed on the right pulmonary artery, and an arteriotomy was performed. The arteriotomy was performed slightly superior to the midline of the pulmonary artery, and the incision was kept smaller than the diameter of the artificial vessel, considering that the pulmonary artery could stretch and the arteriotomy could expand. First, the posterior wall and then the anterior wall were sewn continuously with 7/0 polypropylene suture. Before the last stitch, the air was removed, the knot was tied, and the shunt clamp was opened. In terms of the adequacy of the shunt flow, the increase in saturation, the presence of trills on the shunt, and the decrease in diastolic blood pressure were checked. The desired ideal saturation level was accepted as 80-85%, while FiO2 was 50%. If the saturation levels were stable between targeted ranges, prostaglandin infusion was stopped, and PDA was left open for spontaneous closure. However, if the saturation levels persisted > 90%, the PDA was looped and gradually narrowed with silastic tapes and clips. If the saturation level stabilized > 90% despite PDA narrowing or total occlusion, no additional surgical intervention (shunt narrowing, shunt revision, pulmonary band, etc.) was performed, and the patient was taken to intensive care unit with decongestive treatments. CPB was used in patients who could not tolerate C-clamp occlusion. For shunts to the main pulmonary artery, the technique described by Laks et al.^[6]^ in which aortic anastomosis was performed was used, side-to-side fashion, and the distal part of the graft was occluded with clips.

### Postoperative Monitoring and Treatment

Shunt patency was confirmed by echocardiography in all patients in the first postoperative hours. The total fluid intake of the patients on the first postoperative day was targeted to be 2,000 cc/m^2^. Erythrocyte replacement was performed to keep hematocrit level > 40%. Positive inotropic treatment was titrated to keep mean arterial blood pressure between 35 and 55 mmHg. After surgical bleeding was no longer a problem, unfractionated heparin infusion at 5-10 units/kg was started in all patients, and heparin doses were titrated so that activated clotting time remained > 200 seconds in the measurements performed at three-hour intervals. Additionally, patients were treated with acetylsalicylic acid at a dose of 5 mg/kg on the night of the operation. Patients were started on furosemide infusion if their urine output was < 1 cc/kg/hr. After the 3^rd^ postoperative day, unfractionated heparin infusion was ceased, and low molecular weight heparin was commenced as the anticoagulant treatment. In patients with suspicion of high pulmonary blood flow without significant increase in lactate level or tendency to metabolic acidosis, intensive diuretic and inotrope treatment was continued instead of surgical reintervention.

### Statistical Analysis

Normality assumptions of the variables were examined with the Kolmogorov-Smirnov test. Mean, standard deviation, median, and interquartile range values were used in descriptive statistics of continuous variables. Frequency (n) and percentage (%) values were used to define categorical variables.

Mann-Whitney U test was used to compare continuous variables between both groups. Relationships between categorical variables were examined using Chi-square/Fisher’s exact tests. Logistic regression analysis was used to determine risk factors for in-hospital mortality. The IBM Corp. Released 2017, IBM SPSS Statistics for Windows, version 25.0, Armonk, NY: IBM Corp. software was used in all analyses, and *P*<0.05 was accepted as the significance level.

## RESULTS

Preoperative demographic and clinical data of the patients are shown in Table 1. Of the total 62 patients included in the study, 32 (51.6%) were newborns and 16 (25.8%) had a body weight < 3 kg. The average age of the entire sample was 6.66 ± 10.15 months, and the average body weight was 4.96 ± 2.63 kg. Fourteen (22.6%) patients were premature and five (8%) of the patients had heterotaxy. Urgent shunt surgery was performed on 11 patients (17.7%). While 50% of the patients had biventricular anatomy, the remaining 50% had a single ventricle. MBT shunt was applied to 48 patients (77.4%), while CS was applied to 14 patients (22.6%). Failure of PDA stent implantation occurred in 13 (21%) patients. The stents of eight (12.9%) patients migrated out of the PDA during early follow-up. In the remaining patients, either no endovascular intervention was performed due to unsuitable ductal anatomy, or the procedure was discontinued due to ductal spasm during the stent implantation (8.1%). In-hospital death was observed in 27 (43.5%) patients, and the cause of death could not be clearly determined in five of these patients (18.5%).

**Table 1:** Preoperative demographic and clinical data of the patients.

Parameters	N (%)
Entire cohort	62 (100)
Age (months)	
< 1	32 (51.6)
1-12	20 (32.3)
≥ 12	10 (16.1)
Weight (kg)	
< 3	16 (25.8)
3-5	20 (32.3)
≥ 5	26 (41.9)
Prematurity	14 (22.6)
Heterotaxy	5 (8)
Urgent shunt requirement	11 (17.7)
Preoperative anatomy	
Bi-ventricle	31 (50)
Single ventricle	31 (50)
MBT shunt	48 (77.4)
Central shunt	14 (22.6)
Failure of PDA stent implantation	13 (21)
Cancelled due to ductal spasm	5 (8.1)
Stent malposition	8 (12.9)
Acute shunt thrombosis	4 (6,4)
In-hospital mortality	27 (43.5)
Death with undetermined origin	5 (18.5)

MBT=modified BT; PDA=patent ductus arteriosus

Perioperative variables of the patients who underwent MBT shunt and CS are shown in Table 2. Accordingly, 3 mm shunts were used in three (6.3%) of the MBT shunt patients, but they were not used in any of the CS patients. This may be due to the fact that main pulmonary artery was predominantly used as the outflow vessel, and it was wide enough to implant grafts of 3.5 mm in diameter and above. There was no significant difference between the two surgical procedures in terms of requirement for urgent shunt or CPB, additional simultaneous surgical intervention, need for high postoperative inotropes, in-hospital mortality, preoperative diagnosis, need for crossover shunt, acute shunt occlusion, and progression to Glenn or total correction (*P*>0.05).

**Table 2:** Comparison of perioperative categorical variables between MBT shunt and CS groups.

	Operation				*P*-value
	MBT		CS		
	n	%	n	%	
	48	100	14	100	
Shunt diameter (mm)^*^					-
3	3	6.3	0	0.0	
3.5	13	27.1	5	35.7	
4	23	47.9	5	35.7	
5	9	18.8	4	28.6	
Urgent shunt requirement^**^	9	18.8	2	14.3	1.000
CPB requirement^**^	13	27.1	7	50.0	0.120
Simultaneous surgical intervention in addition to shunt surgery^**^	16	33.3	3	21.4	0.519
High postoperative inotrope requirement^**^	16	33.3	5	35.7	1.000
In-hospital mortality^***^	20	41.7	7	50.0	0.580
Preoperative anatomy^***^					0.544
Bi-ventricle	25	52.1	6	42.9	
Single ventricle	23	47.9	8	57.1	
Acute shunt thrombosis	4	8	0	0	0.578
Progression to Glenn or total correction	28	58.3	7	50.0	0.580

CPB=cardiopulmonary bypass; CS=central shunt; MBT=modified BT

* *P*-value is not given because the assumption of the chi-square test was not met

** Fisher’s exact test;

*** Chi-square test

There was no statistical significant difference between the MBT shunt and CS groups in terms of the preferred shunt size in surgery, patients’ shunt/weight ratio, shunt/target pulmonary artery vessel diameter ratio, total hospital stay (days), and intensive care unit stay (days) (*P*>.05) (Table 3). We believe that it is because we did not perform any shunt operation to the pulmonary arteries < 3.5 mm in diameter and focused on other potential target vessels. This approach was correlated with the shunt/target pulmonary artery vessel diameter ratio being observed < 1 in both surgical procedures.

**Table 3:** Comparison of some operative and postoperative variables between MBT shunt and CS groups.

Parameters	n	Mean ± SD	Median (IQR)	*P*-value
Shunt diameter (mm)				0.679
MBT	48	3.99 ± .57	4.00 (3.50 - 4.00)	
CS	14	4.11 ± .63	4.00 (3.50 - 5.00)	
Shunt/weight (mm/kg)				0.459
MBT	48	1.04 ± 0.58	1.05 (0.63 - 1.28)	
CS	14	0.93 ± 0.44	0.80 (0.60 - 1.15)	
Shunt/target pulmonary artery diameter (mm/mm)				0.859
MBT	48	0.84 ± 0.15	0.88 (0.72 -0.95)	
CS	14	0.83 ± 0.14	0.84 (0.74 - 0.97)	
Total hospital stay (days)				0.448
MBT	48	21.10 ± 24.52	14.00 (7.00 - 24.25)	
CS	14	18.43 ± 10.08	18.00 (11.25 - 23.75)	
Intensive care unit stay (days)				0.584
MBT	48	14.44 ± 16.28	10.00 (3.00 - 17.50)	
CS	14	13.57 ± 9.74	10.50 (6.00 - 20.75)	

CS=central shunt; IQR=interquartile range; MBT=modified BT; SD=standard deviation

Factors affecting in-hospital mortality are examined in Table 4. The rate of congestive heart failure in patients with in-hospital mortality was determined as 66.7% and it was significantly higher than in patients without heart failure (*P*<0.001). In addition, postoperative high pulmonary artery flow was observed in five (18.5%) patients who died, and this data was found to be associated with in-hospital mortality (*P*=0.012). Despite these findings, no significant correlation was found between in-hospital mortality and other variables shown in Table 4 (*P*>0.05).

**Table 4:** Comparison of some categorical variables between patients with and without in-hospital mortality.

	In-hospital mortality				*P*-value
	No		Yes		
	n	%	n	%	
Congestive heart failure^*^	3	8.6	18	66.7	<0.001
High postoperative pulmonary flow^**^	0	0.0	5	18.5	0.012
Infection^*^	12	34.3	7	25.9	0.479
Postoperative bleeding^**^	1	2.9	2	7.4	0.575
Urgent surgery^**^	5	14.3	6	22.2	0.510
Acute shunt occlusion^**^	3	8.6	1	3.7	0.626
CPB requirement^*^	11	31.4	9	33.3	0.874
Preoperative anatomy^*^					0.200
Bi-ventricle	20	57.1	11	40.7	
Single ventricle	15	42.9	16	59.3	
Additional surgical intervention^*^	10	28.6	9	33.3	0.687
PDA stent malposition^**^	5	14.3	8	29.6	0.141
Operation^*^					0.580
MBT	28	80.0	20	74.1	
CS	7	20.0	7	25.9	
Weight (kg)					0.130
< 3	6	17.1	10	37.0	
3-mai.	11	31.4	9	33.3	
≥ 5	18	51.4	8	29.6	
Shunt diameter (mm)^*^					-
3	0	0.0	3	11.1	
3.5	7	20.0	11	40.7	
4	18	51.4	10	37.0	
5	10	28.6	3	11.1	

CPB=cardiopulmonary bypass; CS=central shunt; MBT=modified BT; PDA=patent ductus arteriosus

* Chi-square test;

** Fisher’s exact test;

*** *P*-value is not given because the assumption of the Chi-square test is not met

As a result of the logistic regression analysis (Table 5), it was determined that the development of postoperative congestive heart failure significantly predicted in-hospital mortality (*P*<0.001).

**Table 5:** Factors affecting in-hospital mortality.

Parameters	Wald	Exp(B)	95% CI	95% CI	*P*-value
			Lower limit	Upper limit	
Postoperative congestive heart failure	17.629	21.333	5.113	89.017	<0.001

CI=confidence interval

## DISCUSSION

Despite advanced surgical techniques, improved intensive care services, and state-of-the-art surgical materials, systemic-pulmonary artery shunt operations still have high in-hospital mortality (2.3-16%)^[7]^ and morbidity. This may be due to the fact that not every shunt patient has the same cardiac anatomy and hemodynamics, and the primary pathology of the shunted heart may not always be compatible with the new physiology formed after the shunt surgery. Bove et al.^[8]^ reported 8.7% in-hospital mortality in their retrospective analysis of 150 systemic-pulmonary artery shunt patients. In this study, where the effect of cardiac morphology of patients on mortality was investigated, the highest mortality was in patients with pulmonary atresia/ventricular septal defect (VSD) and single ventricle, with 14% and 13%, respectively. On the other hand, no hospital mortality (0%) was observed in groups such as tetralogy of Fallot, transposition of the great arteries-VSD-pulmonary stenosis, and Ebstein anomaly, which constituted 39% of the entire sample. In addition, it is stated that if shunt complications develop in patients with single ventricle, these patients face a risk of mortality approximately four times higher than other pathologies. Additionally, in patients with a single ventricle, using a shunt with inappropriate diameter for the patient increases the mortality by approximately three times. As a result, patients with single ventricular morphology were found to be associated with high surgical mortality, therefore, the authors recommended that the ductal stenting strategy could be used more frequently in this patient group^[9]^. In our study, the distribution of patients with single ventricle and bi-ventricle was similar, and we could not detect any statistical evidence that this variable had a significant effect on in-hospital mortality.

Acute shunt thrombosis is a life-threatening complication that can lead to in-hospital mortality. It is seen in approximately 9 to 14% of shunted patients^[10,11]^. Risk factors for acute shunt thrombosis after MBT shunt have been meticulously investigated. Some of these can be listed as follows: prematurity^[12]^, low patient weight (< 2.5 kg)^[10,13]^, small target pulmonary artery^[11]^, shunt/pulmonary artery diameter ≥ 0.9^[12]^, shunt/patient weight ≥ 1.3^[10,12,13]^, ineffective anticoagulation^[14]^, errors in surgical technique^[5,10,14]^, surgical approach (thoracotomy *vs.* sternotomy)^[15]^, presence of complex congenital heart disease^[8]^, perioperative hemodynamic instability^[10,14]^, perioperative hypovolemia, presence of main aorticopulmonary collaterals, persistence of antegrade blood flow or PDA following shunt surgery, using 3 mm shunt^[16]^, intraoperative clamping of the shunt, preoperative high hematocrit level^[11]^, and the presence of non-cardiac syndrome^[17]^. Endovascular or surgical revisions after shunt occlusion cause additional morbidity and mortality for patients. Therefore, it is essential to consider every risk factor and approach it accordingly in this surgical procedure. In our sample, acute shunt thrombosis was observed in four patients (6.4%). Three of these patients were in the MBT shunt group and one was in the CS group. All patients underwent urgent surgical reintervention. In the intraoperative evaluation of the patients, it was observed that the diameters of the target pulmonary arteries were all convenient for distal anastomosis. However, technical errors in surgical practice (shunt prepared too long and became twisted, tension in the distal anastomosis, technical error in proximal and distal anastomoses) were detected in all patients. In three of these patients, the MBT shunt was converted to CS, and in one of them, the MBT shunt was reperformed. While two of the patients who underwent conversion to CS survived, one died.

Since shunt-related morbidity remains high, PDA stenting has become a popular alternative method to MBT shunts in PDA-dependent pathologies. Because procedure-related mortality is low (< 1%) and mediumand long-term results are similar to MBT shunt, some centers have adopted the strategy of stent implantation in all PDA-dependent pathologies, regardless of anatomy^[18]^. Despite successful earlyand long-term results, PDA stenting also has serious early complications (9-13%)^[19]^. Development of ductal spasm during the procedure (< 1%) and malposition of the stent (14%) are but a few. If the guide wire can be introduced through the PDA, spasm can be prevented by quickly placing a stent in it. However, in case of stent malposition, it is not always possible to withdraw and reposition the stent. In these cases, the first stent is either fixed using a second stent, or the malposed stent is safely expanded in the distal arterial bed. However, occasionally, the patient’s hemodynamics may deteriorate, and urgent stent removal and shunt surgery may be required. All 62 patients in our study were evaluated for endovascular stenting, and the anatomy of thirteen of them was determined to be suitable for stenting. During the surgery, procedure cancelling occurred in five patients due to persistent PDA spasm, and PDA stent malposition occurred in eight patients. Six of these patients underwent urgent, and two elective, stent removal and/or shunt implantation procedures.

The patient’s weight is typically taken into consideration when determining the diameter of the shunt to be used^[20]^. The general approach is to use 3 mm diameter shunts for patients weighing < 3 kg, 3.5 mm diameter shunts for patients weighing between 3 and 4 kg, and 4 mm diameter shunts for patients weighing > 4 kg. Although this approach is generally useful, in some patients, the pulmonary artery diameter may be too small and incompatible with the patient’s weight. This may lead to the misuse of a larger shunt than should be appropriate. For this reason, aside from the shunt/weight ratio, some researchers also recommend paying attention to the shunt/pulmonary artery ratio and selecting the shunt as this ratio is < 0.9^[10-12,14]^. In shunt implantations above this ratio, no matter how good the anastomosis is, there may be excessive tension on the pulmonary artery wall opposite the anastomosis area. Subsequently, this may cause pulmonary artery distortion, failure of the pulmonary arteries to develop effectively and equally, as well as the development of shunt thrombosis. Our clinical approach was to keep the target pulmonary artery diameter > 3.5 mm. Thus, instead of using a 3 mm shunt to right or left pulmonary arteries < 3.5 mm, our preference was to perform a CS with at least a 3.5 mm PTFE graft to the main pulmonary artery which has a diameter > 3.5 mm. Our aim to follow this principle was to reduce the risk of acute shunt thrombosis that may occur with a 3 mm graft and eliminate the risk of distortion in the target vessel with a 3.5-4 mm graft. As a matter of fact, in line with this principle, we found that the shunt/pulmonary artery diameter ratio was 0.84 and 0.83, respectively, in both our MBT shunt and CS patients. We think that this approach both reduces the risk of surgical technical errors and provides more effective and safe blood flow to the outflow vessel.

Unfortunately, in some patients, pulmonary overcirculation, which has the characteristics of low diastolic blood pressure and systemic and coronary malperfusion, may be encountered due to the selection of a larger graft than required. This may cause the patient to develop acute heart failure secondary to systemic malperfusion, multiorgan failure, lactic acidosis, and coronary ischemia^[21]^. Furthermore, this may require the use of intensive and long-term diuretics and positive inotropes in the postoperative period. This is especially prevalent in patients where arterial inflow is provided directly from the ascending aorta or innominate artery, and the median sternotomy approach is used. Generally, shunt length is short, and shunt is performed to more proximal segments of the pulmonary arteries in these patients. Although this situation can usually be tolerated in patients with biventricular physiology, it has been reported that excessive pulmonary blood flow, that may occur in patients with single ventricular physiology, may have detrimental effects on long-term survival^[15]^. Therefore, it is recommended that shunt selection should lean towards smaller shunts, especially in patients with a single ventricle, and particular attention should be paid to the development of pulmonary overflow and congestive heart failure.

Due to the negative effects of excessive pulmonary blood flow and the development of congestive heart failure on mortality, it is still a matter of debate whether PDA should be closed simultaneously during shunt implantation. Some clinics follow the strategy of controlled spontaneous closure of the ductus arteriosus by ceasing the prostaglandin infusion after shunt implantation^[8,13]^. Thus, in cases of acute shunt occlusion, the patient can be kept alive until emergency surgical intervention is performed. Some authors state that PDA may compete with the shunt for competitive flow and cause thrombosis of the shunt. Therefore, they recommend that PDA should be closed in the same session if SaO2 is ≥ 85-90%, when there is low diastolic blood pressure^[21]^, in order not to disturb the systemic and coronary perfusion. Our clinical approach is to loop the PDA with free silk or a thin vascular tape, instead of primary ligation of the PDA, and narrow it with hemostatic clips, considering the diastolic blood pressure and saturation values. Thanks to this controlled narrowing process, diastolic runoff due to wide PDA can be prevented. In cases with acute shunt occlusion, ductal patency can be restored with prostaglandin infusion until surgical intervention is performed. In cases of shunt-related overcirculation that is not related to PDA flow, it is recommended to stabilize the clinical condition with intensive medical interventions instead of narrowing the shunt^[8]^. This surgical procedure is reported to have high in-hospital mortality^[7]^.

The first CS operation between the ascending aorta and the main pulmonary artery was described in 1962^[22,23]^. With the experience gained in the following years, the advantages and disadvantages of this approach were revealed. In CS that can be performed with median sternotomy, the entire pulmonary arterial bed can be easily accessed and CPB support can be used when necessary. Moreover, since it is centrally located, it can effectively increase the pulmonary flow and be closed quickly and safely after resternotomy. Its most important disadvantage is that it may cause heart failure due to high shunt flow. In order to minimize this complication, it has been suggested that shunt selection should be made depending on the underlying anatomy, status of pulmonary vascular resistance, and the patient’s age. For instance, if the patient is a newborn and has a thickened and small pulmonary artery bed or aortopulmonary collateral arteries with stenotic pulmonary artery segments, a large diameter shunt can be used. On the other hand, if the patient is an infant, body weight should be taken into consideration when choosing a shunt, and small-diameter shunts may be preferred to prevent excessive pulmonary blood flow^[22]^. However, there is still no clear road map in this issue, and the general principle is to use small-diameter shunts in CS.

In our study, we could not detect a significant difference when comparing the shunt diameters, shunt/body weight, shunt/target pulmonary artery diameter ratio, and total intensive care unit and hospitalization rates of MBT shunt and CS patients (Table 3). However, our hospital mortality rate was 43.5%, which was high compared to the literature. When the causes of hospital mortality were examined, it was seen that 66.7% of the patients who died had symptoms of congestive heart failure (Table 4). Moreover, in the regression analysis, this parameter was found to be a risk factor for mortality (*P*<0.01) (Table 5). Postoperative high pulmonary flow was also detected as a risk factor for mortality (*P*=0.012). Although the shunt/body weight and shunt/target pulmonary artery diameter ratios are low, we consider that our high mortality rate may be due to heart failure and multiorgan perfusion disorder due to high pulmonary blood flow.

In our clinic, our principle has always been to shunt the largest target vessel. We think that this approach both prevents the development of acute shunt thrombosis and allows the use of more sophisticated surgical techniques. However, as the result of this study, we noticed that the mortality rate in our sample was higher than in the literature (43.5%). Since we found that the most important reasons for this are congestive heart failure and high pulmonary blood flow, we have planned to change our clinical strategy. Accordingly, as our new strategy, in both MBT shunt and CS, we have started to use smaller shunts and completely close the PDA in all cases with a saturation value > 85%, following shunt implantation. With this strategy, we have aimed to prevent the development of congestive heart failure and to achieve more successful results.

### Limitations

Due to the retrospective, single-center, and non-randomized design of our study, this research is subject to all the inherent limitations associated with retrospective studies. Additionally, the limited number of patients in both shunt groups may have led to the inability to obtain statistically significant results in terms of postoperative mortality caused by morbid factors other than congestive heart failure.

## CONCLUSION

MBT shunt and CS are still frequently used in cyanotic patients today. Use of small-diameter shunts, particularly when centrally located, can prevent the onset of congestive heart failure and lower mortality. Alas, a clear path has not yet been established.
